# Impact of chronic obstructive pulmonary disease on short-term outcome in patients with ST-elevation myocardial infarction during COVID-19 pandemic: insights from the international multicenter ISACS-STEMI registry

**DOI:** 10.1186/s12931-022-02128-0

**Published:** 2022-08-15

**Authors:** Giuseppe De Luca, Matteo Nardin, Magdy Algowhary, Berat Uguz, Dinaldo C. Oliveira, Vladimir Ganyukov, Zan Zimbakov, Miha Cercek, Lisette Okkels Jensen, Poay Huan Loh, Lucian Calmac, Gerard Roura Ferrer, Alexandre Quadros, Marek Milewski, Fortunato Scotto di Uccio, Clemens von Birgelen, Francesco Versaci, Jurrien Ten Berg, Gianni Casella, Aaron Wong Sung Lung, Petr Kala, José Luis Díez Gil, Xavier Carrillo, Maurits Dirksen, Victor M. Becerra-Munoz, Michael Kang-yin Lee, Dafsah Arifa Juzar, Rodrigo de Moura Joaquim, Roberto Paladino, Davor Milicic, Periklis Davlouros, Nikola Bakraceski, Filippo Zilio, Luca Donazzan, Adriaan Kraaijeveld, Gennaro Galasso, Arpad Lux, Lucia Marinucci, Vincenzo Guiducci, Maurizio Menichelli, Alessandra Scoccia, Aylin Hatice Yamac, Kadir Ugur Mert, Xacobe Flores Rios, Tomas Kovarnik, Michal Kidawa, Josè Moreu, Vincent Flavien, Enrico Fabris, Iñigo Lozano Martínez-Luengas, Marco Boccalatte, Francisco Bosa Ojeda, Carlos Arellano-Serrano, Gianluca Caiazzo, Giuseppe Cirrincione, Hsien-Li Kao, Juan Sanchis Forés, Luigi Vignali, Helder Pereira, Stephane Manzo, Santiago Ordoñez, Alev Arat Özkan, Bruno Scheller, Heidi Lehtola, Rui Teles, Christos Mantis, Ylitalo Antti, João A. Brum Silveira, Rodrigo Zoni, Ivan Bessonov, Stefano Savonitto, George Kochiadakis, Dimitrios Alexopoulos, Carlos E. Uribe, John Kanakakis, Benjamin Faurie, Gabriele Gabrielli, Alejandro Gutierrez Barrios, Juan Pablo Bachini, Alex Rocha, Frankie Chor-Cheung Tam, Alfredo Rodriguez, Antonia Anna Lukito, Veauthyelau Saint-Joy, Gustavo Pessah, Andrea Tuccillo, Giuliana Cortese, Guido Parodi, Mohamed Abed Bouraghda, Elvin Kedhi, Pablo Lamelas, Harry Suryapranata, Monica Verdoia

**Affiliations:** 1 Division of Clinical and Experimental Cardiology, AOU Sassari, Sassari, Italy; 2 Third Medicine Division, ASST Spedali Civili, Brescia, Italy; 3 Division of Cardiology, Assiut University Heart Hospital, Assiut University, Asyut, Egypt; 4 Division of Cardiology, Bursa City Hospital, Bursa, Turkey; 5 Pronto de Socorro Cardiologico Prof. Luis Tavares, Centro PROCAPE, Federal University of Pernambuco, Recife, Brazil; 6 Department of Heart and Vascular Surgery, State Research Institute for Complex Issues of Cardiovascular Diseases, Kemerovo, Russia; 7 Medical Faculty, University Clinic for Cardiology, Ss’ Cyril and Methodius University, Skopje, North Macedonia; 8 Centre for Intensive Internal Medicine, University Medical Centre, Ljubljana, Slovenia; 9 Division of Cardiology, Odense Universitets Hospital, Odense, Danemark; 10 Department of Cardiology, National University Hospital, Singapore, Singapore; 11 Clinic Emergency Hospital of Bucharest, Bucharest, Romania; 12 Interventional Cardiology Unit, Heart Disease Institute, Hospital Universitari de Bellvitge, Barcelona, Spain; 13 Instituto de Cardiologia do Rio Grande do Sul, Porto Alegre, Brazil; 14 Division of Cardiology, Medical University of Silezia, Katowice, Poland; 15 Division of Cardiology, Ospedale del Mare, Naples, Italy; 16 Department of Cardiology, Medisch Spectrum Twente, Thoraxcentrum Twente, Enschede, The Netherlands; 17 Division of Cardiology, Ospedale Santa Maria Goretti Latina, Latina, Italy; 18 Division of Cardiology, St Antonius Hospital, Nieuwegein, The Netherlands; 19 Division of Cardiology, Ospedale Maggiore Bologna, Bologna, Italy; 20 Department of Cardiology, National Heart Center, Singapore, Singapore; 21 University Hospital Brno, Medical Faculty of Masaryk University, Brno, Czech Republic; 22 H. Universitario y Politécnico La Fe, Valencia, Spain; 23 Hospital Germans Triasi Pujol, Badalona, Spain; 24 Division of Cardiology, Northwest Clinics, Alkmaar, The Netherlands; 25 Hospital Clínico Universitario Virgen de la Victoria, Málaga, Spain; 26 Department of Cardiology, Queen Elizabeth Hospital, University of Hong Kong, Yau Ma Tei, Hong Kong; 27 Department of Cardiology and Vascular Medicine, University of Indonesia National Cardiovascular Center “Harapan Kita”, Jakarta, Indonesia; 28 Instituto de Cardiologia de Santa Catarina Praia Comprida, São José, Brasil; 29 Division of Cardiology, Clinica Villa dei Fiori, Acerra, Italy; 30 Department of Cardiology, University Hospital Centre, University of Zagreb, Zagreb, Croatia; 31 Invasive Cardiology and Congenital Heart Disease, Patras University Hospital, Patras, Greece; 32 Center for Cardiovascular Diseases, Ohrid, North Macedonia; 33 Division of Cardiology, Ospedale Santa Chiara di Trento, Trento, Italy; 34 Division of Cardiology, Ospedale “S. Maurizio” Bolzano, Bolzano, Italy; 35 Division of Cardiology, UMC Utrecht, Utrecht, The Netherlands; 36 Division of Cardiology, Ospedale San Giovanni di Dio e Ruggi d’Aragona, Salerno, Italy; 37 Maastricht University Medical Center, Maastricht, The Netherlands; 38 Division of Cardiology, Azienda Ospedaliera “Ospedali Riuniti Marche Nord”, Pesaro, Italy; 39 Division of Cardiology, AUSL-IRCCS Reggio Emilia, Reggio Emilia, Italy; 40 Division of Cardiology, Ospedale “F. Spaziani”, Frosinone, Italy; 41 Division of Cardiology, Ospedale “Sant’Anna”, Ferrara, Italy; 42 Department of Cardiology, Hospital Bezmialem Vakıf University, İstanbul, Turkey; 43 Division of Cardiology, Faculty of Medicine, Eskisehir Osmangazi University, Eskisehir, Turkey; 44 Complexo Hospetaliero Universitario La Coruna, La Coruna, Spain; 45 University Hospital Prague, Prague, Czech Republic; 46 Central Hospital of Medical University of Lodz, Lodz, Poland; 47 Division of Cardiology, Complejo Hospitalario de Toledo, Toledo, Spain; 48 Division of Cardiology, Center Hospitalier Universitaire de Lille, Lille, France; 49 Azienda Ospedaliero-Universitaria Ospedali Riuniti Trieste, Trieste, Italy; 50 Division of Cardiology, Hospital Cabueñes, Gijon, Spain; 51 Division of Cardiology, Ospedale Santa Maria delle Grazie, Pozzuoli, Italy; 52 Division of Cardiology, Hospital Universitario de Canarias, Santa Cruz de Tenerife, Spain; 53 Division of Cardiology, Hospital Puerta de Hierro Majadahonda, Madrid, Spain; 54 Division of Cardiology, Ospedale “G Moscati”, Aversa, Italy; 55 Division of Cardiology, Ospedale Civico Arnas, Palermo, Italy; 56 Cardiology Division, Department of Internal Medicine, National Taiwan University Hospital, Tapei, Taiwan; 57 Division of Cardiology, Hospital Clinico Universitario de Valencia, Valencia, Spain; 58 Interventional Cardiology Unit, Azienda Ospedaliera Sanitaria, Parma, Italy; 59 Cardiology Department, Hospital Garcia de Orta, Pragal, Almada, Portugal; 60 Division of Cardiology, CHU Lariboisière, AP-HP, Paris VII University, INSERM UMRS 942, Paris, France; 61 Instituto Cardiovascular de Buenos Aires, Buenos Aires, Argentina; 62 Cardiology Institute, Istanbul University, Istanbul, Turkey; 63 Division of Cardiology Clinical and Experimental Interventional Cardiology, University of Saarland, Saarbrücken, Germany; 64 Division of Cardiology, Oulu University Hospital, Oulu, Finland; 65 Division of Cardiology, Hospital de Santa Cruz, CHLO-Nova Medical School, CEDOC, Lisbon, Portugal; 66 Division of Cardiology, Kontantopoulion Hospital, Athens, Greece; 67 Division of Cardiology, Heart Centre Turku, Turku, Finland; 68 Division of Cardiology, Hospital de Santo António, Porto, Portugal; 69 Department of Teaching and Research, Instituto de Cardiología de Corrientes “Juana F. Cabral”, Corrientes, Argentina; 70 Tyumen Cardiology Research Center, Tyumen, Russia; 71 Division of Cardiology, Ospedale “A. Manzoni”, Lecco, Italy; 72 Iraklion University Hospital, Crete, Greece; 73 Division of Cardiology, Attikon University Hospital, Athens, Greece; 74 Division of Cardiology, Universidad UPB, Universidad CES, Medellin, Colombia; 75 Division of Cardiology, Alexandra Hospital, Athens, Greece; 76 Division of Cardiology, Groupe Hospitalier Mutualiste de Grenoble, Grenoble, France; 77 Interventional Cardiology Unit, Azienda Ospedaliero Universitaria“Ospedali Riuniti”, Ancona, Italy; 78 Division of Cardiology, Hospital Puerta del Mar, Cadiz, Spain; 79 Instituto de Cardiologia Integral, Montevideo, Uruguay; 80 Department of Cardiology and Cardiovascular Interventions, Instituto Nacional de Cirugía Cardíaca, Montevideo, Uruguay; 81 Department of Cardiology, Queen Mary Hospital, University of Hong Kong, Pok Fu Lam, Hong Kong; 82 Division of Cardiology, Otamendi Hospital, Buenos Aires, Argentina; 83 Cardiovascular Department Pelita, Harapan University/Heart Center Siloam Lippo Village Hospital, Tangerang, Banten Indonesia; 84 Center Hospitalier d’Antibes Juan Les Pins, Antibes, France; 85 Division of Cardiology, Hospiatl Cordoba, Cordoba, Argentina; 86 Department of Statistical Sciences, University of Padova, Padua, Italy; 87 Department of Cardiology, ASL 4 Liguria, Lavagna, Italy; 88 Division of Cardiology, Blida University Hospital, Blida, Algeria; 89 Division of Cardiology, Hopital Erasmus, Universitè Libre de Bruxelles, Brussels, Belgium; 90 Division of Cardiology, Radboud University Medical Center, Nijmegen, The Netherlands; 91 Division of Cardiology, Ospedale degli Infermi, ASL Biella, Ponderano, Italy; 92 University of Sassari, Sassari, Italy

**Keywords:** STEMI, COPD, Mortality

## Abstract

**Background:**

Chronic obstructive pulmonary disease (COPD) is projected to become the third cause of mortality worldwide. COPD shares several pathophysiological mechanisms with cardiovascular disease, especially atherosclerosis. However, no definite answers are available on the prognostic role of COPD in the setting of ST elevation myocardial infarction (STEMI), especially during COVID-19 pandemic, among patients undergoing primary angioplasty, that is therefore the aim of the current study.

**Methods:**

In the ISACS-STEMI COVID-19 registry we included retrospectively patients with STEMI treated with primary percutaneous coronary intervention (PCI) between March and June of 2019 and 2020 from 109 high-volume primary PCI centers in 4 continents.

**Results:**

A total of 15,686 patients were included in this analysis. Of them, 810 (5.2%) subjects had a COPD diagnosis. They were more often elderly and with a more pronounced cardiovascular risk profile. No preminent procedural dissimilarities were noticed except for a lower proportion of dual antiplatelet therapy at discharge among COPD patients (98.9% vs. 98.1%, P = 0.038). With regards to short-term fatal outcomes, both in-hospital and 30-days mortality occurred more frequently among COPD patients, similarly in pre-COVID-19 and COVID-19 era. However, after adjustment for main baseline differences, COPD did not result as independent predictor for in-hospital death (adjusted OR [95% CI] = 0.913[0.658–1.266], P = 0.585) nor for 30-days mortality (adjusted OR [95% CI] = 0.850 [0.620–1.164], P = 0.310). No significant differences were detected in terms of SARS-CoV-2 positivity between the two groups.

**Conclusion:**

This is one of the largest studies investigating characteristics and outcome of COPD patients with STEMI undergoing primary angioplasty, especially during COVID pandemic. COPD was associated with significantly higher rates of in-hospital and 30-days mortality. However, this association disappeared after adjustment for baseline characteristics. Furthermore, COPD did not significantly affect SARS-CoV-2 positivity.

Trial registration number: NCT 04412655 (2nd June 2020).

## Introduction

Chronic obstructive pulmonary disease (COPD) has been estimated affecting more than 200 million people worldwide, representing the fourth highest cause of death in the world, with a projection to be the third leading cause of death in next years [1]. It represents a chronic inflammatory process affecting airways and lung parenchyma leading to an irreversible or partly reversible airflow obstruction [2]. Main risk factor is constituted by cigarette smoking, that is also crucial in promoting atherosclerosis. From a pathological point of view COPD and atherosclerosis share a pro-inflammatory environmental, conditioning cardiovascular system by endothelial dysfunction and increased platelet activation: impairment of the equilibrium in vessel homeostasis promotes plaque development and instability which are further aggravated by enhanced platelet reactivity.

This is reflected on the clinical side, where COPD patients are recognized at higher risk of cardiovascular diseases and mortality [3, 4]. Moreover, up to 17% of patients admitted for an acute myocardial infarction is affected by COPD [5]. Previous investigations showed potential negative impact of COPD on outcome of patients undergoing percutaneous coronary intervention (PCI) [6, 7], even if scant results were reported in the specific setting of ST-elevation myocardial infarction (STEMI), that deserves a fast mechanical reperfusion with primary PCI [8]. Contrasting findings have been described in relation to COPD impact on short-term mortality in STEMI patients, without conclusive sentence [9, 10]. In addition, most of prior evidence has  been achieved a remarkable number of years ago, with a potential reduced validity nowadays, given the improvements in the management of acute cardiovascular diseases. Furthermore, last year was characterized by the outbreak of Coronavirus disease 2019 (COVID-19) pandemic, that deeply impacted in the world health care systems. Alongside the systemic involvement of severe acute respiratory syndrome coronavirus 2 (SARS-CoV-2) infection, lungs constitute the main involved organs with clinical manifestations ranging from mild flu-like symptoms to acute respiratory distress syndrome [11]. COPD makes patients more prone to respiratory tract infection, especially of viral etiology, and it was demonstrated conferring higher probability of poor outcomes in COVID-19 patients [12].

Therefore, we aimed to inquire from a large, contemporary, multicenter registry of STEMI patients if COPD diagnosis at hospital admission could be deemed a risk factor of adverse outcome, especially during COVID-19 pandemic.

## Methods

### Study design and population

Study population is constituted by patients enrolled in the ISACS-STEMI COVID-19 (NCT 04412655), a retrospective multicenter registry including STEMI patients enrolled by 109 high-volume primary PCI centers from Europe, Latin America, South-East Asia and North-Africa. This study was conducted to compare STEMI patients treated from March 1^st^ until June 30^th^ of 2019 with those admitted within the same period of 2020. We included patients treated within 48 h from symptoms onset. We did not exclude those patients undergoing mechanical revascularization after failed thrombolysis (rescue angioplasty).

Collected baseline characteristics included demographic, clinical, procedural data, data on total ischemia time, door-to-balloon time, referral to primary PCI facility and PCI procedural data. COPD diagnosis was determined at admission according to international guidelines society [2]. The outcomes of interest were all-cause in-hospital mortality and all-cause mortality at 30 days. Follow-up data were obtained from outpatients' visit records or by telephone call.

The study was approved by the Ethical Committee of AOU Maggiore della Carità, Novara. Detailed data have previously been provided [13, 14].

### Statistics

All analyses were performed by using SPSS Statistics Software 23.0 (IBM SPSS Inc., Chicago, Illinois). Patients were grouped according to COPD diagnosis. Absolute frequencies and percentages were used for categorical variables. Conversely, continuous variables were reported using median and interquartile range. Normal distribution of continuous variables was tested by the Kolmogorov–Smirnov test. Chi-square test was adopted for categorical variable, while ANOVA or Mann–Whitney test, as appropriate, were used for continuous variables.

Multivariable logistic regression analyses were performed to identify the association of COPD with in-hospital and 30-day mortality and SARS-CoV-2 infection after adjustment for baseline confounding factors. All significant variable (set at a *P*‐value < 0.1) were entered in block into the model. A P < 0.05 was considered statistically significant. The data coordinating center was established at the Eastern Piedmont University, Novara, Italy.

## Results

A total of 15,686 patients admitted for STEMI and undergoing mechanical reperfusion were included. Of them, 810 (5.2%) patients were affected by COPD, while 14,876 (94.2%) patients were not. Table 1 summarizes baseline features of the two groups. COPD patients displayed a higher cardiovascular risk profile than non-COPD. In particular, they were older, active smokers, more frequently affected by hypertension, diabetes mellitus, hypercholesterolemia, with a history of prior STEMI and PCI. A higher percentage of COPD patients compared to non-COPD was from Europe with consequent lower fraction from South East Asia and North Africa. Differences neither in referral to hospital for primary PCI, nor in time delays were detected between COPD and non-COPD patients, that were more frequently admitted for anterior STEMI but less often in cardiogenic shock.

**Table 1:** Baseline demographic and clinical characteristics according to COPD diagnosis

Variables	COPD (n = 810)	Non COPD (n = 14,876)	P-value
Age, years—median, IQR)	68 (61–76)	62 (54–71)	< 0.001
Age > 75 year—n. (%)	229 (28.3)	2711 (18.2)	< 0.001
Male gender—n. (%)	603 (74.4)	11,269 (75.8)	0.398
Diabetes mellitus—n (%)	235 (29.0)	3526 (23.7)	0.001
Hypertension—n (%)	545 (67.3)	8095 (54.4)	< 0.001
Hypercholesterolemia—n (%)	412 (50.9)	5824 (39.2)	< 0.001
Active smoker—n (%)	567 (70.0)	7963 (53.5)	< 0.001
Family history of CAD—n (%)	150 (18.5)	3013 (20.3)	0.231
Previous STEMI—n (%)	127 (15.7)	1374 (9.2)	< 0.001
Previous PCI—n (%)	160 (19.8)	1781 (12.0)	< 0.001
Previous CABG—n (%)	20 (2.5)	242 (1.6)	0.068
*Geographic area*			< 0.001
Europe n (%)	725 (89.5)	11,692 (78.6)	
Latin-America—n (%)	61 (7.5)	1289 (8.7)	
South East Asia—n (%)	21 (2.6)	1272 (8.6)	
North Africa—n (%)	3 (0.4)	623 (4.2)	
*Referral to primary PCI hospital*			0.388
Ambulance (from community)—n (%)	383 (47.5)	7136 (48.0)	
HUB—n (%)	215 (26.5)	4175 (28.1)	
Spoke—n (%)	210 (25.9)	3565 (24.0)	
*Time delays*			
Ischemia time, minutes—median (IQR)*	210 (129–370)	210 (121–379)	0.786
Total Ischemia time > 12 h—n (%)	89 (11.0)	1527 (10.3)	0.510
Door-to-balloon time, minutes—median (IQR)*	40 (26–60)	40 (25–70)	0.709
Door-to-balloon time > 30 min (%)—n (%)	506 (62.5)	9102 (61.2)	0.465
*Clinical presentation*			
Anterior STEMI—n (%)	335 (41.4)	6940 (46.7)	0.003
Out-of-hospital cardiac arrest—n (%)	48 (5.9)	908 (6.1)	0.953
Cardiogenic shock—n (%)	83 (10.2)	1081 (7.3)	0.007
Rescue PCI for failed thrombolysis—n (%)	50 (6.2)	1049 (7.1)	0.340

*COPD*  chronic obstructive pulmonary disease, *CAD*  coronary artery disease, *STEMI*  ST-segment elevation myocardial infarction, *PCI*  percutaneous coronary intervention, *CABG 0* coronary artery bypass graft

^*^Mann–Whitney test

Angiographic and procedural characteristics are summarized in Table 2. No differences in stenting and use of drug-eluting stent were found, as well as in post-procedural TIMI 3 flow, Gp IIb-IIIa inhibitors, Cangrelor and Bivalirudin use. A numerically higher proportion of non-COPD patients received dual antiplatelet therapy after the procedure compared to COPD patients (98.9% vs. 98.1%, P = 0.038), while no difference was present regarding the use renin angiotensin system inhibitors during the hospitalization (56.8% vs. 54.7%, respectively, P = 0.232).

**Table 2:** Angiographic and procedural characteristics according to COPD diagnosis

Variables	COPD (n = 810)	Non COPD (n = 14,876)	P-value
Radial access (%)	648 (80.0)	1456 (77.0)	0.048
*Culprit vessel*			0.009
Left main—n (%)	17 (2.1)	224 (1.5)	
LAD—n (%)	328 (40.5)	6872 (46.2)	
Circumflex—n (%)	151 (18.6)	2134 (14.3)	
RCA—n (%)	308 (38.0)	5532 (37.2)	
Anterolateral branch—n (%)	2 (0.2)	39 (0.3)	
SVG—n (%)	4 (0.5)	75 (0.5)	
In-stent thrombosis—n (%)	48 (5.9)	560 (3.8)	0.002
Multivessel disease—n (%)	426 (52.6)	7371 (49.5)	0.239
Preprocedural TIMI 0 flow—n (%)	513 (63.3)	9967 (67.0)	0.031
Thrombectomy—n (%)	151 (18.6)	2412 (16.2)	0.069
Stenting—n (%)	753 (93.0)	13,647 (91.7)	0.456
Drug-eluting stent—n (%)	730 (90.1)	13,163 (88.5)	0.153
Postprocedural TIMI 3 flow—n (%)	745 (92.0)	13,692 (92.0)	0.954
Gp IIb–IIIa inhibitors/Cangrelor—n (%)	189 (23.3)	3050 (20.5)	0.053
Bivalirudin—n (%)	2 (0.2)	33 (0.2)	0.883
Mechanical support—n (%)	32 (4.0)	459 (3.1)	0.169
*Additional PCI*			0.584
During the index procedure—n (%)	75 (9.3)	1305 (8.8)	
Staged—n (%)	94 (11.6)	1583 (10.6)	
DAPT therapy—n (%)	795 (98.1)	14,717 (98.9)	0.038

*COPD* chronic obstructive pulmonary disease, *LAD*  left anterior descending, *RCA*  right coronary artery, *SVG*  saphenous vein graft, *TIMI*  thrombolysis in myocardial infarction, *DAPT*  dual antiplatelet therapy

A similar percentage of SARS-CoV-2 positive patients between the two groups was recognized (1.0% vs 0.7% in COPD and non-COPD patients, respectively, P = 0.162).

**Fig. 1:**
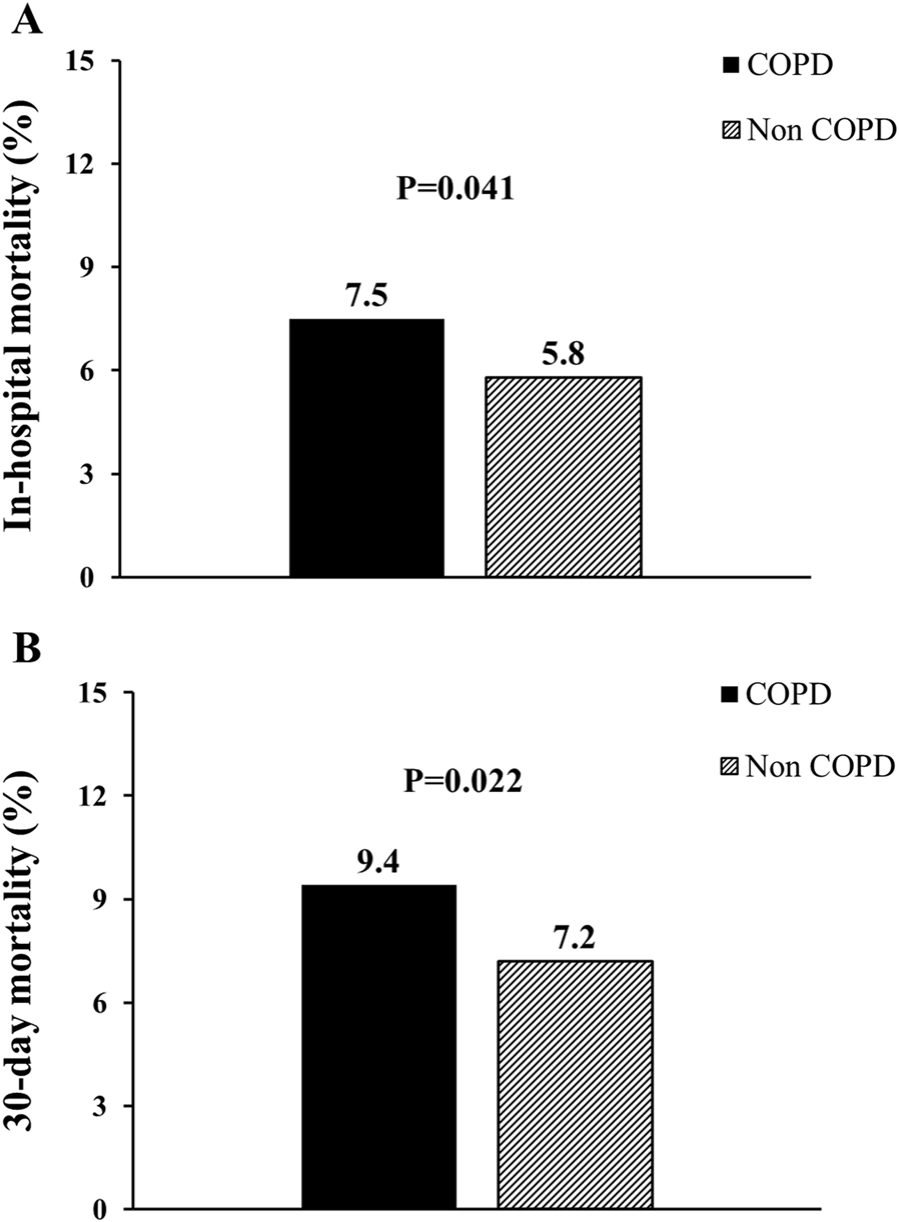
Bar graphs show in-hospital (panel **A**) and 30-day (panel **B**) mortality in the overall cohort, according to COPD diagnosis. *COPD*  chronic obstructive pulmonary disease

**Fig. 2:**
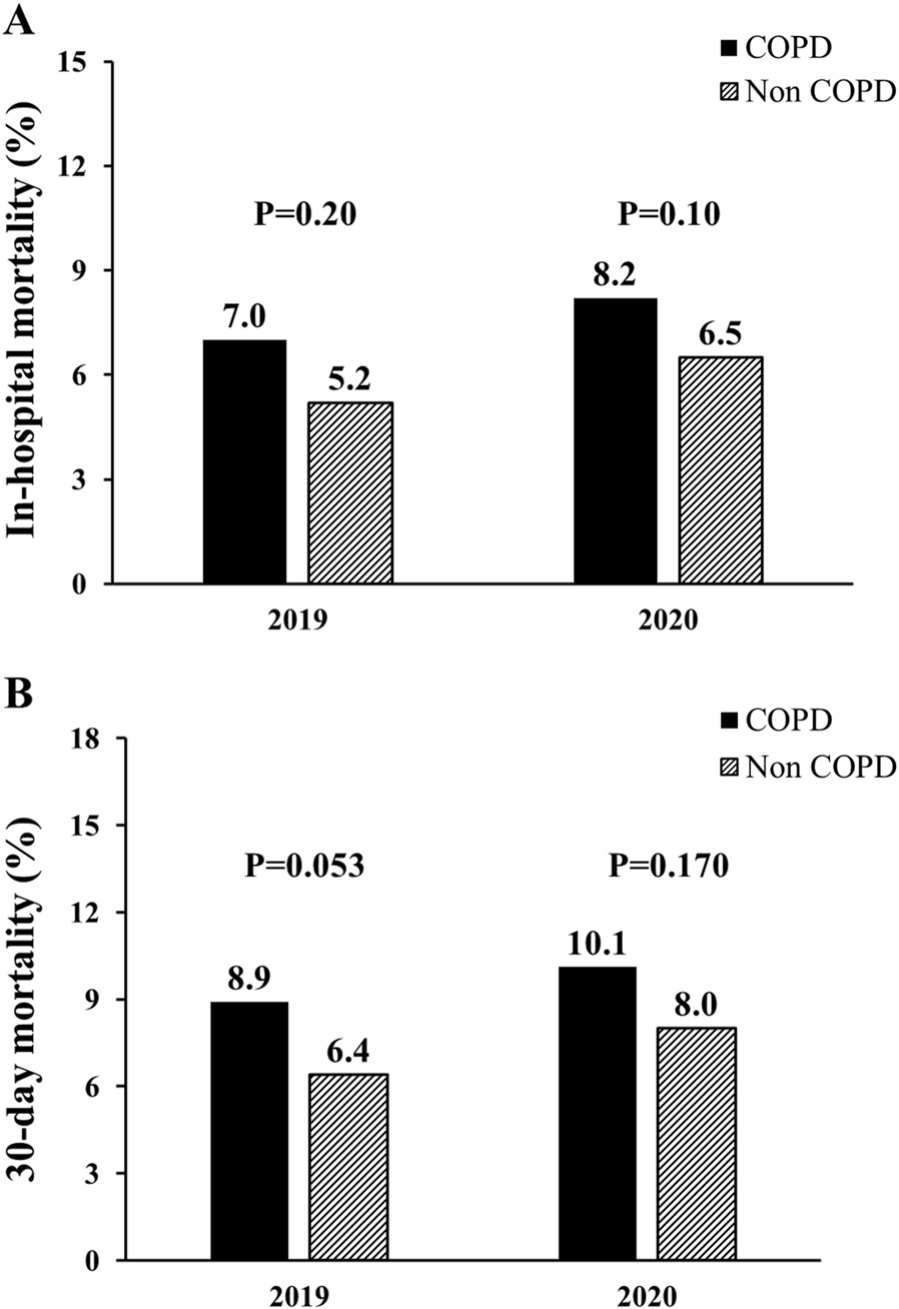
Bar graphs show in-hospital (panel **A**) and 30-day (panel **B**) mortality in 2019 and in 2020 cohorts, according to COPD diagnosis. *COPD*  chronic obstructive pulmonary disease

**Fig. 3:**
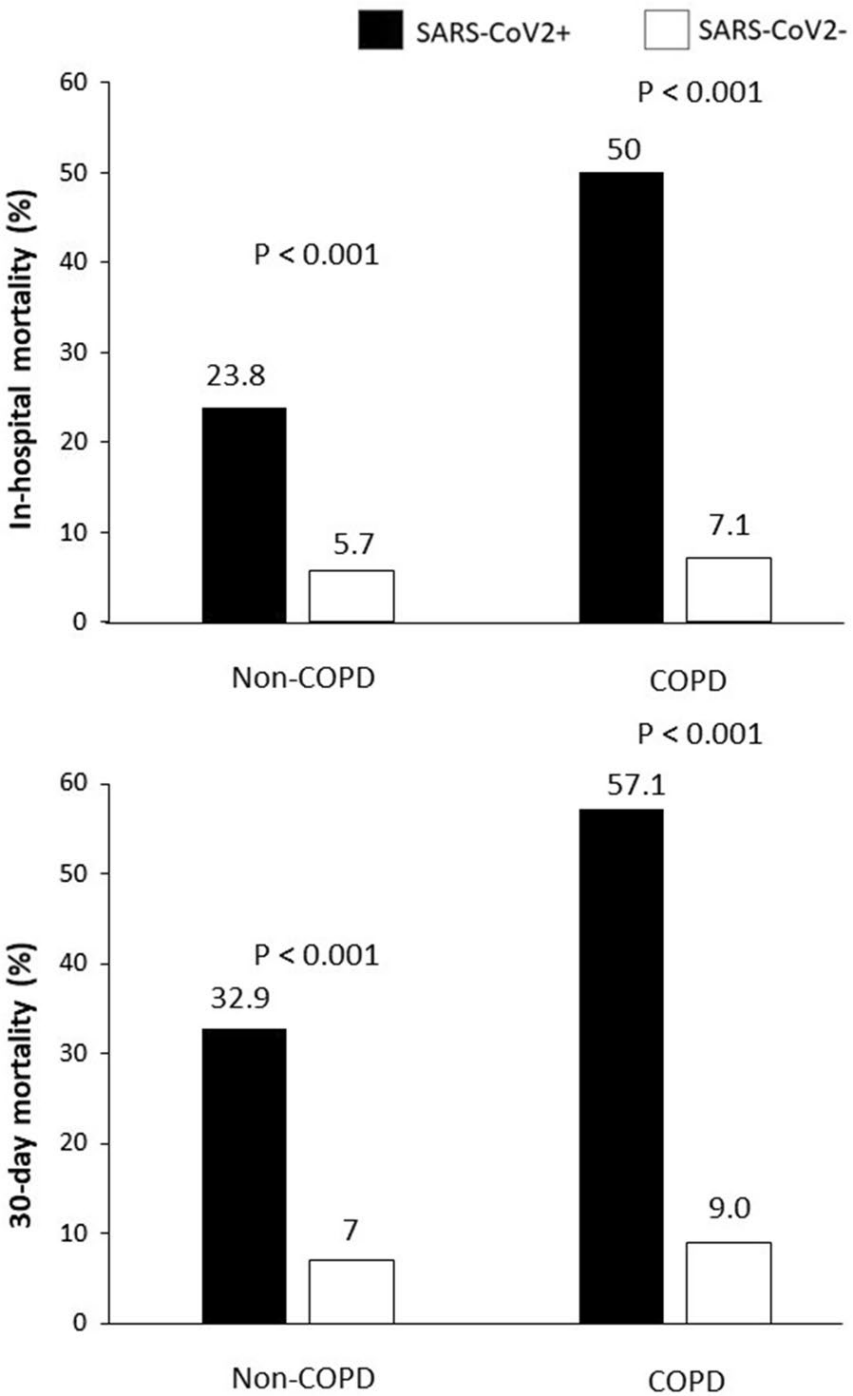
Bar graphs show the impact of SARS-CoV-2 positivity on in-hospital and 30-day mortality in both COPD and non-COPD patients. COPD = chronic obstructive pulmonary disease

A significant higher rate of mortality was experienced by COPD patients than ones without, both considering in-hospital (7.5% vs 5.8%, respectively, P = 0.041) and 30-day (9.4% vs 7.2%, respectively, P = 0.022) mortality (Fig. 1). Similar results were observed when a separate analysis was conducted in 2019 (pre-COVID) (in-Hospital mortality: 7% vs 5.2%, p = 0.20; 30-day mortality: 8.9% vs 6.4%, p = 0.053) and 2020 (COVID-19 pandemic) (in-hospital mortality 8.2% vs 6.5%, p = 0.10; 30-day mortality: 10.1% vs 8%, p = 0.170) (Fig. 2). SARS-Cov-2 positivity was associated with a significantly higher in-hospital and 30 mortality in both COPD and non-COPD patients (Fig. 3).

The detrimental relationship between COPD and short-term mortality was not confirmed after the adjustment for all the confounding baseline and procedural characteristics (age > 75 years, diabetes, active smoking, hypertension, hypercholesterolemia, previous STEMI, previous PCI, geographic area, cardiogenic shock, anterior MI, radial access, in-stent thrombosis, pre-procedural TIMI flow, dual antiplatelet therapy), neither for in-hospital (adjusted OR [95% CI] = 0.91 [0.66–1.27], P = 0.58) nor for 30-day mortality (adjusted HR [95% CI] = 0.85 [0.62–1.16], P = 0.31).

## Discussion

Main findings from our large, multicenter, contemporary registry of STEMI patients are consistent with a low but not negligible percentage of patients with COPD. They displayed a significant higher baseline cardiovascular risk profile that portended an increase rate of in-hospital and short-term mortality. However, after adjustment for baseline clinical confounders, COPD did not result an independent predictor of fatal outcomes.

The strict relationship between lung and heart is well known, even if the mechanisms of this interplay are less intuitive and not fully delineated, especially on the pathological side: of course, sharing two key risk factors, age and cigarette smoking, enforces the link between COPD and ischemic heart disease. However, during last years the paradigm of COPD as localized inflammatory disease has shifted to systemic involvement, driven by the evidences of higher level of C-reactive protein, fibrinogen and pro-inflammatory cytokines [15, 16]. Together with oxidative stress and the consequent endothelial dysfunction and arterial stiffness, COPD presence meaningfully contributes to atherosclerotic disease progression. Moreover, plaque stability is altered by enhanced levels of matrix metalloproteinases, and the concomitant higher platelet activation favors thrombus formation [17, 18].

Results from clinical studies have shown a prevalence of coronary artery disease among COPD patients ranging from 10 to 38% [19, 20] with enhanced cardiovascular mortality following an episode of acute exacerbation [21]. Furthermore, as outlined by Campo et al., among COPD patients referred for an acute exacerbation, the increased risk of adverse cardiac events at follow-up was even worse in patients without history of ischemic heart disease [22].

Similarly, individuals with acute myocardial infarction are more prone to adverse outcomes if a concomitant COPD diagnosis was present [23]. Data from a large registry in the United Kingdom, after correction for other cardiovascular risk factor, suggests increased risk of mortality among COPD patients compared to ones without after an acute myocardial infarction [24] The same authors however underscored an appreciable reduction, even still significant, of the risk of mortality for both STEMI and Non-STEMI, after adjustment for confounders. In this way, Serban et al. argued that the increase of mortality after a MI in COPD patients was more likely due to non-optimal medical therapy rather than disease-specific impact [25], and Andell et al. underscored the potential delay in revascularization treatment as additional detrimental issue in COPD patients [26].

Further distinctions have been enlightened by prior studies with regards of STEMI or Non-STEMI presentation. Enriquez et colleagues have shown no significant independent role of COPD into prediction of in-hospital mortality among STEMI patients, whilst it was exclusive of among those with Non-STEMI [27]. Moreover, Lazzeri et al. have found in more than 800 STEMI patients no impact of COPD in short-term mortality at multivariate analysis [10]. Reasons of discrepancies in literature findings could be sought in the fact that most of prior studies was conducted before the 2010 [23], thus earlier than last improvements achieved in primary PCI for STEMI patients [28–30]. Updated data defining the role and impact of COPD in contemporary large cohort of patients admitted for STEMI are therefore lacking.

In our large multicenter cohort of patients admitted for STEMI a relatively limited proportion of subjects diagnosed with COPD. Their enhanced cardiovascular risk of COPD patients was made-up by more frequent co-presence of active smoking, diabetes mellitus, hypertension, hypercholesterolemia, prior acute cardiac ischemic event, in line with previous reports on the topic [31]. Moreover, advanced age and presentation with cardiogenic shock portrayed patients with COPD compared to those without, while no differences in cardiac arrest as clinical presentation was detected, similarly to prior studies [32].

COPD patients were more prone to fatal outcomes displaying both for in-hospital and 30-day mortality a higher percentage that patients without COPD. However, these significant differences were not confirmed at multivariate logistic regression analyses that did not show an independent role of COPD diagnosis to predict short-term mortality. Our findings are inserted in the controversy still persistent on whatever COPD might represent a risk factor unassociated with others or if it reflects the burden of comorbidities linked to this condition [25, 33]. Additional issues raised by other authors concerned the treatment provided to COPD patients with acute MI: in fact, the part of COPD patients with STEMI displaying the lower risk of adverse outcomes was identified being on appropriate treatment for the lung disease [34]. Also Stefan et al. reported that COPD patients still continue to receive less frequent evidence-base therapy after acute MI, despite signals of amelioration are noticeable across years [5]. In our cohort we did not appreciate any differences in term of use rates of renin angiotensin system inhibitors, cornerstone of drug therapy after STEMI [35].

Additional value of this investigation is the crucial information regarding the ongoing COVID-19 pandemic impact, especially considering our inspection of a lung disease. COPD is known providing higher risk of infection: lung inflammation, airflow obstruction, and impaired clearance of respiratory virus are elements supporting a greater risk for COVID-19 and its severity among COPD patients [2, 36–38]. Data from a large meta-analysis confirmed an increased odds of adverse outcomes, including mortality among COVID-19 with already diagnosed COPD compared to ones without [12]. Also Aveyard et al. have reported COPD patients at higher risk of severe COVID-19 disease, even if the mortality rate for SARS-CoV-2 infection compared to other causes, was not significant in young adults, while in older COPD patients cardiovascular and other causes consistently have contributed the most to mortality [39]. Notably, in our cohort, after inquiring separately data of 2019 and 2020, the worse outcome of COPD patients was similarly observed in both groups, even though the adverse outcomes differences did not reach the significance. Main explanation concerns the reduced statistical power after splitting patients in the two cohorts according to the year. In addition, with respect of SARS-CoV-2 positivity, we reported similar, low rates between patients with and without COPD.

Findings from our large multicenter registry provide an updated snapshot of the impact of COPD on short term outcomes in the acute setting of STEMI. Based on our data, COPD does not represent itself an independent negative prognostic determinant. Therefore, COPD should not be used for risk stratification and COPD patients should not be object of more intensive and aggressive therapies as compared to the standard care for STEMI patients. Dedicated, prospective studies may offer definite and comprehensive answers about the COPD contribution on mortality after an acute MI.

### Limitations

Our findings should be viewed in light of some limitations. The retrospective non-randomized design of our study precludes to definite answer on the impact of COPD on morality in patients experiencing a STEMI. Not all variable of interest could have been collected, reducing the allowed investigations.

The diagnosis of COPD was based on medical records of every patients, and systematic confirmation through pulmonary function tests in case of suspect of doubt diagnosis was not performed. As well, we were not able to classify COPD patients according to their inflammatory endotype [40], that have surely provided more comprehensive findings on relationship between COPD and STEMI.

The ISACS-STEMI registry was not conceived to examine the role of COPD, therefore data are missing on complete in-hospital and post-discharge drug treatment regimens, specifically regarding the COPD, preventing analyses on essential aspect of the treated topic [25], even if the high number of patients included, more than 10-times of the majority of prior investigations, can make the findings pretty confident.

## Conclusions

This is one of the largest studies investigating characteristics and outcome of COPD patients with STEMI undergoing primary angioplasty, especially during COVID-19 pandemic. COPD was associated with significantly higher rates of in-hospital and 30-days mortality. However, this association disappeared after adjustment for baseline characteristics. Furthermore, COPD did not significantly affect SARS-CoV-2 positivity.

## Data Availability

The datasets used and/or analyzed during the current study are available from the corresponding author on reasonable request.

## Abbreviations

COPD: Chronic obstructive pulmonary disease
COVID-19: Coronavirus disease—2019
PCI: Percutaneous coronary intervention
SARS-CoV-2: Severe acute respiratory syndrome coronavirus 2
STEMI: ST-elevation myocardial infarction
